# Cross-talk between energy and redox metabolism in astrocyte-neuron functional cooperation

**DOI:** 10.1042/EBC20220075

**Published:** 2023-03-03

**Authors:** Angeles Almeida, Daniel Jimenez-Blasco, Juan P. Bolaños

**Affiliations:** 1 Instituto de Biologia Funcional y Genomica (IBFG), Universidad de Salamanca, CSIC, Salamanca, Spain; 2 Instituto de Investigacion Biomedica de Salamanca (IBSAL), Hospital Universitario de Salamanca, Universidad de Salamanca, CSIC, Salamanca, Spain; 3 Centro de Investigación Biomédica en Red de Fragilidad y Envejecimiento Saludable (CIBERFES), Madrid, Spain

**Keywords:** animal welfare, astrocytes, metabolic coupling, neurons, redox homeostasis

## Abstract

Astrocytes show unique anatomical, morphological, and metabolic features to take up substrates from the blood and metabolize them for local delivery to active synapses to sustain neuron function. In the present review, we specifically focus on key molecular aspects of energy and redox metabolism that facilitate this astrocyte-neuronal coupling in a controlled manner. Basal glycolysis is co-ordinated by the anaphase-promoting complex/cyclosome (APC/C)-Cdh1, a ubiquitin ligase that targets the proglycolytic enzyme 6-phosphofructokinase-2,6-bisphosphastate-3 (PFKFB3) for degradation. APC/C-Cdh1 activity is more robust in neurons than in astrocytes, which determine that PFKFB3 abundance and glycolytic rate are weaker in neurons. The low PFKFB3 activity in neurons facilitates glucose-6-phosphate oxidation via the pentose-phosphate pathway, which promotes antioxidant protection. Conversely, the high PFKFB3 activity in astrocytes allows the production and release of glycolytic lactate, which is taken up by neurons that use it as an oxidizable substrate. Importantly, the mitochondrial respiratory chain is tighter assembled in neurons than in astrocytes, thus the bioenergetic efficiency of mitochondria is higher in neurons. Because of this, the production of reactive oxygen species (mROS) by mitochondrial complex I is very low in neurons and very high in astrocytes. Such a naturally occurring high abundance of mROS in astrocytes physiologically determines a specific transcriptional fingerprint that contributes to sustaining cognitive performance. We conclude that the energy and redox metabolism of astrocytes must complementarily match that of neurons to regulate brain function and animal welfare.

## Introduction

The high-energy supply required for neuronal activity has been classically related to the use of glucose as the brain’s main energy substrate [1,2], along with the consumption of almost 20% of inhaled O_2_ [3]. These high-energy costs depend closely on metabolic involvement with astrocytes for energy control and redox homeostasis [4,5], given their role as essential partners for neurotransmission and behavior [6,7]. To do this, astrocytes form a syncytium by establishing cellular processes to contact blood capillaries with neuronal soma and synapses. Together with abundant gap junctions, such processes account for an abundant exchange of intermediates that cover the energetic and metabolic demands in the nervous system [2]. This astrocyte-neuron metabolic coupling is clearly observed during glutamatergic neurotransmission, where astrocytes efficiently take up neuronal-derived glutamate from the synaptic space through Na^+^-dependent active transporters [8]. Therefore, glutamate uptake is energy-costly for astrocytes and occurs at the expense of ATP, used to restore the Na^+^ gradient by the Na^+^/K^+^ ATPase pump [9,10]. In astrocytes, intracellular glutamate may follow at least two distinct metabolic fates, its conversion to α-ketoglutarate for oxidation within mitochondria through the tricarboxylic acid cycle (TCA), or its conversion in glutamine by glutamine synthetase, absent from neurons, to be released and taken up by neighboring neurons, which convert it back to glutamate by glutaminase. Importantly, the activation of the Na^+^/K^+^ ATPase pump is paralleled by an increase in glucose uptake [11,12] and coupled with glycolysis in astrocytes [13].

Brain glycolysis is mainly regulated by the E3 ubiquitin ligase anaphase-promoting complex/cyclosome (APC/C)-Cdh1, which targets the key regulatory glycolytic enzyme 6-phosphofructo-2-kinase/fructose-2,6-bisphosphastate-3 (PFKFB3) for proteasomal degradation [14,15]. In contrast with neurons, the low APC/C-Cdh1 activity in astrocytes triggers PFKFB3 stabilization and a high glycolytic activity to provide lactate as a bioenergetic substrate to neurons [14]. These findings have been confirmed worldwide by many laboratories, both in primary cells and *in vivo*, and their importance in health and disease have been established [16–20]. Conversely, the mitochondrial respiratory chain is tighter assembled in neurons than in astrocytes. Thus, neurons depend mainly on the mitochondrial oxidative phosphorylation (OXPHOS) to fulfil their energy demands, while astrocytes mostly rely on glycolysis for energy production [21–24]. Nevertheless, astrocytic mitochondria are important organelles both for energy and signaling purposes [25]. Consequently, the redox homeostasis in both neurons and astrocytes is different, although they are tightly coupled to sustain neurotransmission. Moreover, astrocytes provide antioxidant defence to neurons, which is essential to prevent neurodegeneration [24]. Thus, the bioenergetics and redox cooperation between neurons and astrocytes regulate neuronal survival, brain function, and animal welfare, which might provide new therapeutic targets to fight against neurodegenerative diseases.

## Glycolysis is complementarily regulated in astrocytes and neurons

**Figure 1:**
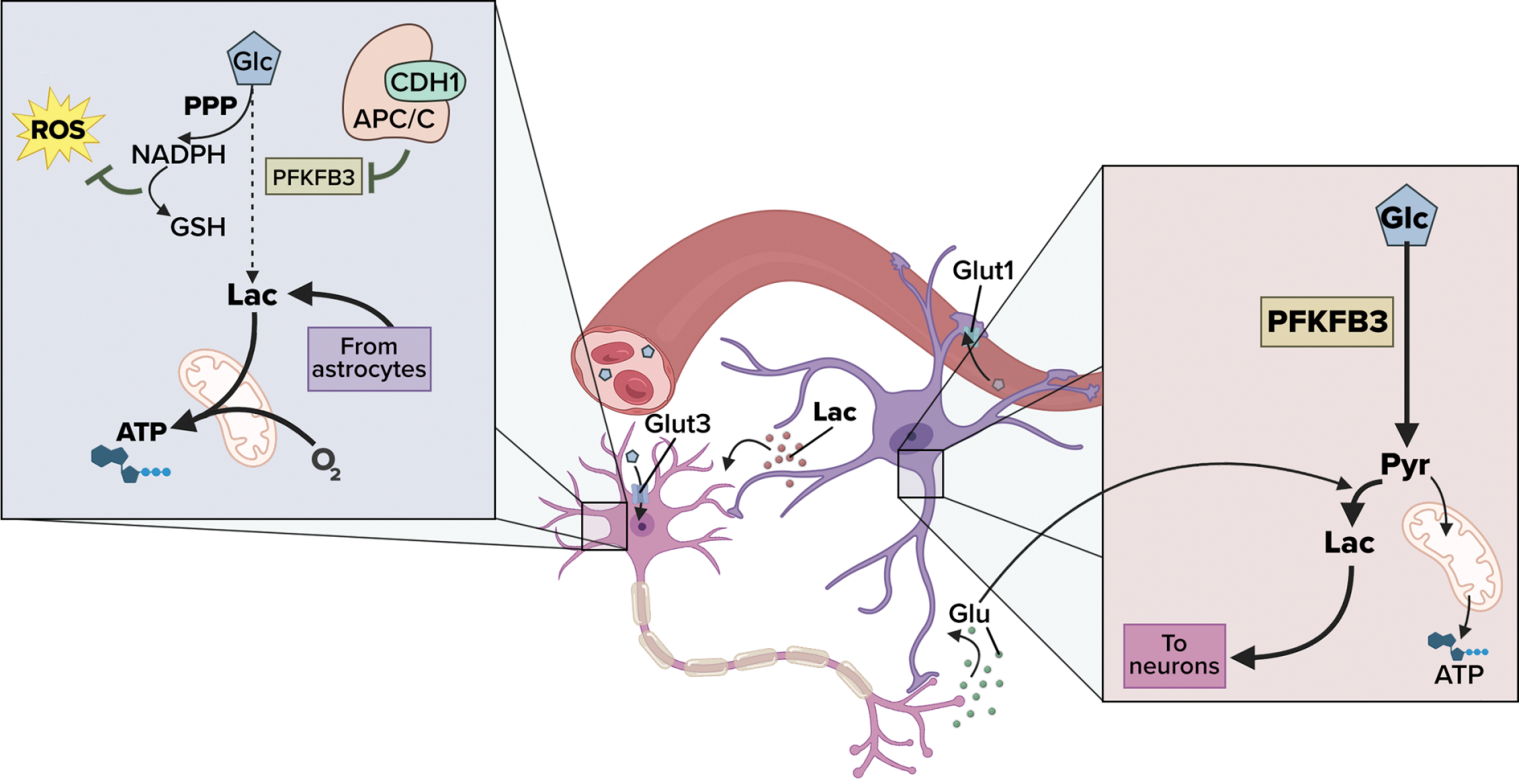
The metabolism of astrocytes matches that of neurons Glycolysis is regulated, amongst other factors, by the ubiquitin ligase APC/C-Cdh1, which targets the proglycolytic enzyme PFKFB3 for proteasomal degradation in neurons. In astrocytes, APC/C-Cdh1 activity is low, allowing PFKFB3 stabilization and higher glycolysis. In neurons, low glycolysis allows glucose conversion into PPP to sustain antioxidant protection, whereas in astrocytes, high glycolysis releases lactate that may be used by neurons.

Glycolysis is mainly regulated by the enzymatic activities of hexokinase, 6-phosphofructo-1-kinase (PFK1), and pyruvate kinase (PK). In astrocytes, PFK1-specific activity is approximately fourfold than found in neurons, and the levels of PFK1 powerful allosteric activator, fructose-2,6-bisphosphate (F2,6P_2_) is twofold [14]. F2,6P_2_ synthesis is controlled in astrocytes mainly by the isoform 3 of the PFKFB enzyme (PFKFB3) [14]. Interestingly, PFKFB3 has a high, approximately 700-fold kinase versus bisphosphatase ratio [26]. Hence, PFKFB3 levels are directly proportional to F2,6-P_2_-synthesizing activity. PFKFB3 knockdown abolishes the ability of astrocytes to up-regulate glycolysis upon mitochondrial inhibition, suggesting that PFKFB3 is important to maintain the glycolytic phenotype of astrocytes [14]. However, PFKFB3 is virtually absent from neurons, because of the continuous destabilization by the ubiquitin-proteasome pathway [24,27] (Figure 1). Thus, only the PFKFB3 isoform contains a ^142^Lys-Glu-Asn (*KEN*) *box* that targets it for ubiquitination by APC/C-Cdh1 [27], which is an E3 ubiquitin ligase known for its role in the regulation of mitosis, meiosis [28], tumor suppression, and genome stability [29]. APC/C-Cdh1 regulates important functions in neurons, such as axonal growth [30–32], cortical neurogenesis [33], and survival [34–36]. In cortical neurons, Cdh1 knockdown leads to PFKFB3 accumulation, which is sufficient to increase glycolysis [27]. This was the first observation to describe a role for a cell cycle-related protein (APC/C-Cdh1) in metabolism, mimicked by PFKFB3 full-length cDNA overexpression [27]. In contrast, Cdh1 protein levels and APC/C-Cdh1 ubiquitylating activity are very low in astrocytes, which explains their high levels of PFKFB3 and glycolytic activity [27]. In neurons, the index of glucose that is oxidized in the TCA cycle after having been converted into pyruvate, analyzed as the rate of [6-^14^C] glucose incorporated into ^14^CO_2_, is negligible when compared with astrocytes [27,37]. In addition, glycolysis, assessed as the rate of ^3^H_2_O formation from [3-^3^H] glucose and thus accurately reflecting the flux of glucose through glycolysis [38], is approximately four- to fivefold slower in neurons than in astrocytes [27]. It seems, therefore, that the neuronal capacity to perform glycolysis is limited. Interestingly, the overactivation of glutamatergic receptors, which inhibits APC/C-Cdh1 via cyclin-dependent kinase-5 (Cdk5)-p25 [35], stabilizes PFKFB3 and other substrates, as cyclin B1 and Rock2 [39], causing neuronal metabolic switch and apoptotic death [35,36]. Altogether, these results suggest that full activation of glycolysis is dangerous for neurons. In contrast, glycolysis flux is strongly up-regulated in astrocytes, either upon inhibition of mitochondrial respiration by, e.g., nitric oxide or cyanide via 5’-AMP-activated protein kinase (AMPK)-mediated activation of PFKFB3 [14,23], or upon nitric oxide-dependent hypoxia-inducible factor-1α (HIF1α) activation, which causes enhanced expression of most glycolytic enzymes including PFKFB3 [40].

## Neuronal function depends on astrocytic glucose metabolism

Astrocytes store glycogen [41], which, after glycogenolysis, serves to supply lactate as an energy substrate for neurons; in contrast, lactate oxidative utilization is comparatively less efficient in astrocytes [42]. Astrocytes lack the mitochondrial aspartate/glutamate carrier, which reduces the ability of these cells to use the malate/aspartate shuttle to transfer NADH-reducing equivalents to mitochondria as a mechanism to recover cytosolic NAD^+^. Therefore, in these cells, the pyruvate-to-lactate conversion becomes a predominant system to regenerate the cytosolic NAD^+^ via lactate dehydrogenase isoform-5 (LDH5), needed to keep active the glycolytic flux [41]. Neurons, in contrast, are more efficient cells to converting lactate to pyruvate through the LDH1 isoform. Altogether, these metabolic differences between astrocytes and neurons help explaining their adaption to couple their metabolism and to transferring lactate from the glial to the neuronal compartment [41], sustaining the bases for the astrocyte-neuron lactate shuttle (ANLS) model [9,43] (Figure 1). *In vivo* evidence for the occurrence of ANLS [44] strongly suggests that it constitutes an evolutionarily well-preserved mechanism of neuronal survival [45]. Importantly, altered ANLS that occurs with dysfunctional monocarboxylate transporter-4 and -2 (MCT4 and MCT2), responsible for the lactate release from astrocytes or uptake by neurons, and lactate content in the brain has been described in Alzheimer’s disease [2,46,47]. Thus, memory formation requires energy, which is provided by learning-dependent regulations of glucose metabolism pathways [48–51]. Interestingly, glucose metabolism shifts as the brain matures [18]. While the adult hippocampus preferentially employs glycogenolysis and astrocyte-neuronal lactate-mediated coupling, the juvenile hippocampus selectively requires, in addition to higher astrocyte-neuronal lactate transport, a direct neuronal glucose transport and a functional PFKFB3 in neurons [18]. Furthermore, the expression of APC/C-Cdh1 in juvenile hippocampal neurons was inverse when compared with adult neurons [18]. This substantial increase in neuronal glucose metabolism may explain the significantly higher levels of excitability, synaptogenesis, and synapse pruning during development [52] and may protect them from death that could be otherwise caused by their high rate of oxidative phosphorylation [18].

## Redox control of neurons depends on their own glucose metabolism

The phenomenon of neurotransmission not only is a highly energetically expensive process but it is associated with a high production of reactive oxygen species (ROS) derived from different sources, which generally involves Ca^2+^ influx and glutamatergic stimulation [4,53,54]. For this reason, the antioxidant systems of neurons must be effective in dealing with these high levels of ROS to which they are subjected. To do this, unlike astrocytes, neurons derive a substantial proportion of glucose consumption through the pentose phosphate pathway (PPP), responsible for regenerating the levels of NADPH necessary for the efficient reduction in glutathione (GSH), the most abundant antioxidant in the brain [24,27,38]. GSH is mostly synthesized in astrocytes by two steps, catalyzed by glutamate-cysteine ligase (GCL) and glutathione synthetase (GSS) [55,56]. Neurons also use this biosynthetic machinery to resynthesize GSH, although with a lower abundance, by capturing amino acid precursors resulting from the degradation of astrocytic GSH [55–57]. The loss of a robust antioxidant capacity in neurons is related to the cognitive impairment associated with certain neurodegenerative diseases that concur with the loss of GSH [58,59]. Interestingly, a proportion of Parkinson’s disease (PD) patients harbor gene mutations that encode for the PTEN-induced kinase 1 (PINK1)-Parkin axis, which is critical to regulate mitophagy [60]. Loss of PINK1 activity induces ROS that may lead to the stabilization of HIF1α, a master up-regulator of glycolysis [61]. While high glycolytic rates are related to increased growth and proliferation of astrocytes in patients with PD [62], a greater glycolytic flow in neurons leads to a detriment of the PPP and reduced GSH [27]. This metabolic switch could help explaining neuronal death in PD and other neurodegenerative diseases coursing with impairment mitophagy, such as Batten disease [16]. Given the need to maintain proper GSH levels to preserve neuronal function, increasing GSH concentration may therefore be a strong strategy to provide neuroprotection [63,64]. This occurs, even if complete conversion to GSH is not achieved, and its immediate precursor, γ-glutamylcysteine can still be used by glutathione peroxidase (GPx) to confer protection against neuronal loss and motor impairment [65].

## The astrocyte-neuron glutathione shuttle couples neurotransmission with neuronal antioxidant protection

The master regulator of the antioxidant response in the brain is the transcriptional activator of nuclear erythroid-related factor 2 (Nrf2) [66–69]. Nrf2 governs the transcription of a wide spectrum of antioxidant enzymes, not only those required for the GSH pathway, such as GCL and GPx, but other enzymes such as heme oxygenase-1 (HO-1), thioredoxin (Trx), NAD(P)H dehydrogenase quinone (Nqo-1), and enzymes involved in NADPH regeneration [70–72]. The poor antioxidant response and increased susceptibility of neurons to ROS are largely due to the continued degradation and destabilization of Nrf2 by the Cullin3-Kelch-like ECH-associated protein 1 (Cul3-KEAP1) complex. Under basal conditions, Nrf2 binds to the redox sensor KEAP1, which, in the absence of ROS, allows the interaction of Nrf2 with E3 ubiquitin ligase Cul3, for its polyubiquitination and subsequent proteasomal degradation. After the accumulation of ROS, the oxidation of key cysteine residues in KEAP1 takes place to induce a conformational change in the Cul3-KEAP1 complex, allowing nuclear translocation and transcriptional activity of Nrf2 [72,73]. Here, Nrf2 induces the expression of genes whose promoters contain its binding site, referred to as the antioxidant response element (ARE) [74]. In neurons, levels of Nrf2 decline by a mechanism involving the epigenetic repression of the Nrf2 promoter leading to histone hypoacetylation around the Nrf2 transcription start site, making these cells especially vulnerable to ROS [75]. However, Nrf2 is more stabilized in astrocytes making them primarily responsible for the removal of ROS in the nervous system, since antioxidants are not only mainly synthesized in astrocytes but have also been shown to supply them to adjacent neurons in both *in vitro* and *in vivo* models [70,76]. For this reason, astrocytes are key to providing antioxidant capacity to neurons, although neurons are also able to induce the expression of antioxidant genes in an Nrf2-independent manner through the interpretation of Ca^2+^ signals [77–79].

In astrocytes, the activation of glutamatergic receptors (GluR), mainly N-methyl-D-aspartate receptors (NMDAR), triggers a signal transduction pathway involving phospholipase C mediated by the release of Ca^2+^ from the endoplasmic reticulum and the subsequent activation of protein kinase Cδ (PKCδ). The activation of this kinase promotes, by phosphorylation, the stabilization of p35, a cofactor of Cdk5. In the cytosol, the active Cdk5-p35 complex phosphorylates Nrf2 on the residues Thr^395^, Ser^433^, and Thr^439^, which is enough to promote the translocation of Nrf2 to the nucleus and induce the expression of the antioxidant machinery [70]. This astrocytic pathway provides an antioxidant reserve that neurons use for ROS detoxification. The increase in intracellular Ca^2+^ produced, following excessive activation of NMDARs by glutamate, is known as an excitotoxic response that could lead to neuronal death when unresolved [35,80]. The neuronal activity involves an astrocyte response that entails, among other antioxidant mechanisms, *de novo* biosynthesis of GSH and its release to neurons through the astrocyte-neuron glutathione shuttle (ANGS) [24]. Therefore, this intercellular communication via NMDAR couples neurotransmission with neuronal survival, as demonstrated during ischemic preconditioning [81].

## The mitochondrial energy efficiency of astrocytes impacts on redox signaling and organismal welfare

**Figure 2:**
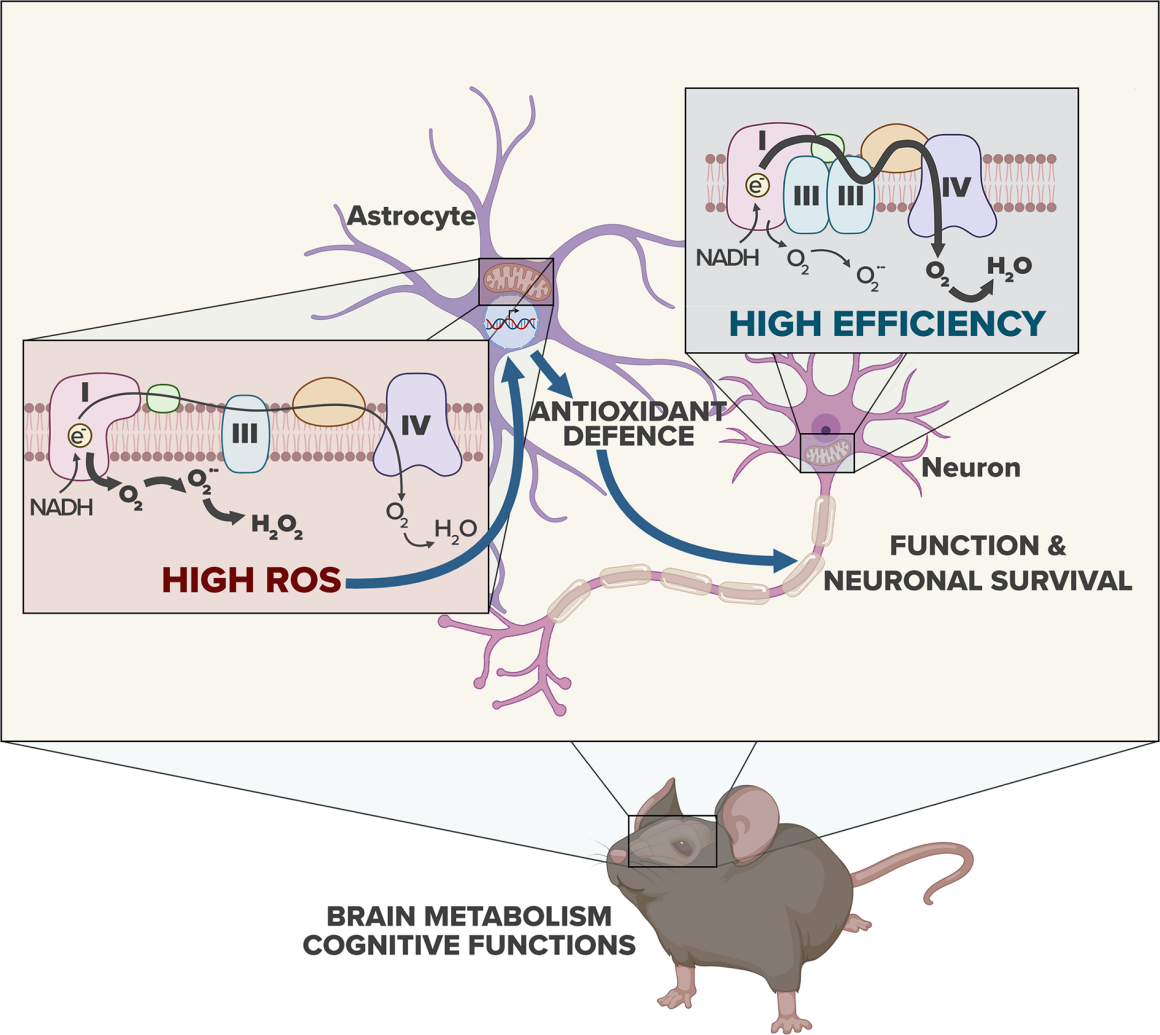
Astrocytic mitochondrial ROS physiologically regulates metabolism and behavior The mitochondrial respiratory chain is poorly assembled in astrocytes, which determines a low bioenergetic efficiency, but a high mitochondrial ROS (mROS) production. The high abundance of mROS in astrocytes actively promotes a transcriptional program that contributes to sustaining energy and redox metabolism, impacting on mouse cognitive performance. In contrast, the higher assembly of the mitochondrial respiratory chain in neurons determines higher energy efficiency and lower mROS production in these cells.

In recent years, numerous findings have changed the current view of how neural cells produce ROS and what are their function within the nervous system. Despite being considered deleterious by-products, ROS have now been shown to be essential for proper metabolic and redox coupling. To understand this issue, we should look at the regulation of the OXPHOS. One of the main differential characteristics between neurons and astrocytes is that neurons depend mainly on OXPHOS to fulfil their energy requirements, while astrocytes mainly rely on glycolysis for energy production [21–24]. Mitochondria are considered the main producers of ROS within the cell [82]. Thus, along with other enzymatic sources within mitochondria [83], increased ROS production emerges from electron transfer through mitochondrial complexes during OXPHOS [82]. To account for an efficient function of the electron transport chain (ETC) and ATP production from respiration, respiratory complexes are assembled into quaternary structures called respiratory supercomplexes (RSCs) [84,85]. Surprisingly, astrocytes barely assemble CI to CIII and CIV to form RSCs, presenting most of the CI disassembled. However, neurons have most of their complexes assembled in RSC, housing CI, CIII, and CIV [25,85] (Figure 2). The less active, free CI of astrocytes, accounts for less efficient respiration along with a significant increase in ROS production [25]. In free CI, the flavin mononucleotide containing the NDUFV1 subunit is more available to interact with O_2_ [85]. In this scenario, the generation of superoxide anion (O_2_^·−^) is carried out since the ETC is in an oxidized state [86], with a low efficiency to consume O_2_ from NADH substrates in astrocytes, compared with neurons. In a model of targeted expression of catalase to the mitochondria, astrocytic ROS was attenuated *in vivo* and was shown to regulate brain metabolism, neuronal function, and organismal behavior [87]. Moreover, the impact of astrocytic mitochondrial ROS on behavior was also confirmed in a different biological environment. Thus, the production of astrocytic mitochondrial ROS can be reduced *in vivo* by the activation of cannabinoid-1 receptors that are present in mitochondria (mtCB1) [88]. This decrease in astrocytic mitochondrial ROS, by down-regulating HIF1α, attenuates the glycolytic flow and lactate release to neurons, causing neuronal bioenergetic deficit and social impairment [88]. Therefore, these concepts are in line with hormesis, defined as the process of adaptive response to a persistent, but not lethal, stressful stimulus that generates in the cell resistance to such stress, probably due to a greater antioxidant capacity [89–91]. This fact could explain how astrocytes are prepared to efficiently manage oxidative stress and how redox homeostasis constitutes a clear example of compartmentalization of neural cells that is closely related to brain metabolism [87].

## Summary

- Ubiquitin ligase APC/C-Cdh1 co-ordinates astrocyte-neuron metabolic coupling by promoting PFKFB3 destabilization and attenuation of glycolysis in neurons, facilitating PPP activity for antioxidant protection.
- In astrocytes, APC/C-Cdh1 activity is very low resulting in PFKFB3 stabilization and higher basal glycolytic rate and lactate release.
- The mitochondrial respiratory chain is tighter assembled in neurons than in astrocytes, which determines higher bioenergetic efficiency and lower mROS production in the neurons.
- The weaker assembly of the mitochondrial respiratory chain in astrocytes determines lower bioenergetic efficiency and higher mROS production in these cells.
- Astrocytic mROS promote a transcriptional signature that contributes to sustaining the metabolic and redox metabolism and brain function.

## Abbreviations

AMPK: 5′-AMP-activated protein kinase
ANGS: astrocyte-neuron glutathione shuttle
ANLS: astrocyte-neuron lactate shuttle
APC/C: anaphase-promoting complex/cyclosome
ARE: antioxidant response element
CB1: cannabinoid-1 receptor
Cdk5: cyclin-dependent kinase-5
Cul3: Cullin3
ETC: electron transport chain
F2,6P_2_: fructose-2,6-bisphosphate
GCL: glutamate-cysteine ligase
GluR: glutamatergic receptor
GPx: glutathione peroxidase
GSH: reduced glutathione
GSS: glutathione synthetase
HIF1α: hypoxia-inducible factor-1α
HO-1: heme oxygenase-1
KEAP1: Kelch-like ECH-associated protein 1
LDH: lactate dehydrogenase
MCT: monocarboxylate transporter
mROS: mitochondrial reactive oxygen species
NMDA: N-methyl-D-aspartate
Nqo-1: NAD(P)H dehydrogenase quinone
Nrf2: nuclear erythroid-related factor 2
OXPHOS: mitochondrial oxidative phosphorylation
PD: Parkinson’s disease
PFK1: 6-phosphofructo-1-kinase
PFKFB3: 6-phosphofructo-2-kinase/fructosa-2,6-bisphosphastate-3
PINK1: PTEN-induced kinase 1
PK: protein kinase
PPP: pentose phosphate pathway
RSC: respiratory supercomplex
TCA: tricarboxylic acid cycle
Trx: thioredoxin
